# Mediterranean-style diet reduces metabolic syndrome components in obese children and adolescents with obesity

**DOI:** 10.1186/1471-2431-14-175

**Published:** 2014-07-05

**Authors:** Lubia Velázquez-López, Gerardo Santiago-Díaz, Julia Nava-Hernández, Abril V Muñoz-Torres, Patricia Medina-Bravo, Margarita Torres-Tamayo

**Affiliations:** 1 Clinical Epidemiology Research Unit, Hospital General Regional No. 1 Carlos Macgregor Sánchez-Navarro, Instituto Mexicano del Seguro Social, Mexico City, Mexico; 2 Centro Médico Nacional la Raza, Instituto Mexicano del Seguro Social, Mexico City, Mexico; 3 Public Health Department, Universidad Nacional Autónoma de México, Mexico City, Mexico; 4 Endocrinology Department, Hospital Infantil de México Federico Gómez, Secretaría de Salud (SSA), Mexico City, Mexico; 5 Community Health Research Unit, Hospital Infantil de México Federico Gómez, Secretaría de Salud (SSA), Mexico City, Mexico

**Keywords:** Obesity, Metabolic syndrome, Mediterranean diet, Children, Adolescents

## Abstract

**Background:**

The beneficial effects of the Mediterranean diet have been amply proven in adults with cardiovascular risk factors. The effects of this diet have not been extensively assessed in pediatric populations with obesity, insulin resistance (IR) and metabolic syndrome (MetS). The aim of this study was to assess the efficacy of the Mediterranean style diet (MSD) to decrease cardiovascular risk factors in children and adolescents with obesity.

**Methods:**

Participants were randomly assigned to a MSD rich in polyunsaturated fatty acids, fiber, flavonoids and antioxidants (60% of energy from carbohydrate, 25% from fat, and 15% from protein, (n = 24); or a standard diet (55% of carbohydrate, 30% from fat and 15% from protein, (n = 25), the caloric ingest was individualized. At baseline and 16-week of intervention, the glucose, triglycerides (TG), total cholesterol (TC), HDL-C, LDL-C were measured as well as the body composition and anthropometric data. The diet compliance was determined by the 24-hour recalls.

Paired Student’s t and Macnemar’s test were used to compare effects in biochemical, body composition, anthropometric, and dietary variables.

**Results:**

The MSD group had a significantly decrease in BMI, lean mass, fat mass, glucose, TC, TG, HDL-C and LDL-C. (p < 0.05); the diet compliance increased consumption of omega 9 fatty acids, zinc, vitamin E, selenium, and decreased consumption of saturated fatty acids (p < 0.05). The standard diet group decrease in glucose levels and frequency of glucose >100 mg/dL (p < 0.05).

**Conclusion:**

The MSD improves the BMI, glucose and lipid profile in children and adolescents with obesity and any MetS component.

## Background

Over the last three decades, there has been a documented increase in the prevalence of pediatric obesity worldwide [1]. In Mexico, the 2006 National Survey of Health and Nutrition reported a 26.8% combined prevalence of overweight and obese children aged 5 to 11. In 2012, the national combined prevalence of overweight and obese children aged 5 to 11 was 34.4% (19.8 and 14.6%, respectively), whereas 35% of adolescents were overweight or obese [2].

As previously reported, the predicting factors that are most tightly linked to metabolic syndrome (MetS) are obesity and insulin resistance (IR). A study reported that 90% of obese adolescents present with at least one MetS component, whereas 30% meet all MetS criteria [3], including abdominal obesity, dyslipidemia (increase in triglycerides [TG] and low high-density lipoprotein cholesterol [HDL-C] levels), high blood pressure and glucose intolerance [4,5]. The importance and interest in MetS lies in its association with type 2 diabetes mellitus (T2DM), coronary heart disease, and increased mortality, even in subjects without T2DM [6].

The beneficial effects of poly- and monounsaturated fats on the recommended indexes for omega-3 (ω3) have been determined to result in decreased obesity and IR [7]. Additionally, the association between the glycemic index (GI) and glycemic load (GL) with Homeostatic Model Assessment of IR index (HOMA-IR) has been established, as well as the prevalence of MetS when foods with a high GI are consumed. The beneficial effect of high-fiber cereals and whole grains has also been demonstrated [8].

A diet rich in soluble fiber (20 g/1000 kcal) and low in polyunsaturated fats (20% of total calories) and a decreased consumption of food items with a high GI, can decrease the prevalence of MetS by improving blood pressure (BP) and IR [9,10]. Weight loss improves insulin sensitivity and decreases visceral fat content. The level of oxidative stress is increased in patients with MetS, causing an endogenous and exogenous depletion of antioxidant reserves. Patients with MetS do not consume enough fruit and vegetables, so it is necessary to increase their intake to meet vitamin C and carotenoid quotas [11].

Over the last few decades, the Mediterranean diet has been shown to decrease cardiovascular events and increase life expectancy in adult populations [12-14].

Similarly, it has been shown to efficiently decrease MetS by 20-43%, regardless of age, sex, physical activity, lipid levels, and BP [15]. This type of diet characteristically uses olive oil, both as a dressing and to cook food, and contains fish, wheat, olives, and grapes [16,17].

The beneficial effects of the Mediterranean diet have been proven. Similarly, in combination with dried fruits, its main component, olive oil, has been shown to decrease total cholesterol (TC) and TG levels [18,19].

The effect of a Mediterranean-style diet (MSD) has not been extensively assessed in pediatric populations presenting with cardiovascular risk factors. Thus, the objective of the present study was to assess the efficacy of nutritional therapy using an MSD to decrease MetS indicators in obese children and adolescents.

## Methods

An open-label study was conducted in 49 children and adolescents attending the family medicine unit at the Mexican Social Security Institute. The subjects were selected from a multicentric study in Mexico called “Prevention and early treatment of T2D in a pediatric population”. Subjects meeting the following criteria were selected: Body Mass Index (BMI) ≥95^th^ percentile and any MetS component, according to modified International Diabetes Federation (IDF) criteria for children and adolescents; waist circumference (WC) ≥90^th^ percentile; fasting glucose ≥100 mg/dL; TG ≥150 mg/dL; HDL-C ≤ 40 mg/dL; systolic blood pressure (SBP) ≥130 mmHg and/ or diastolic blood pressure (DBP) ≥85 mmHg [20]. On beginning the study, none of the participating candidates presented any chronic illness, nor were they receiving pharmacological treatment for obesity and/or its comorbidities that would limit their participation in the dietetic intervention. This research was conducted in accordance with the Declaration of Helsinki, 59th WMA General Assembly, Seoul, Republic of Korea, October 2008 for research involving human subjects. Ethical approval was obtained from the Research Ethics Committee at the Carlos Macgregor Sanchez Navarro Hospital, Mexico City. Written consent was provided by all participants and their parents before enrollment.

Clinical history was obtained and a complete examination was carried out for all participants. The presence of acanthosis nigricans in the neck and armpits was assessed. SBP and DBP were measured three times with a 5 min interval between measurements, with the patient having remained seated for more than 5 min. The arterial pressure value was determined from the average of the last two measurements. Anthropometric measurements were recorded by nutritionists and standardized using the Habicht method according to the specifications recommended by Lohman et al. [21,22].

Weight and height were measured using a TANITA™ scale (model TBF-215), which provides information on fat percentage, fat mass, and lean body mass in kilograms using bioelectrical impedance of the lower segments. WC was measured after determining the midpoint between the last rib and the upper edge of the iliac crest on the right-hand side. Hip circumference was determined at the widest point of the trochanters. Both measurements were taken three times, and the average values of the second and third measurements were used for the analysis.

TC and TG concentrations were measured by enzymatic methods. HDL-C was quantified after precipitation of lipoproteins containing apoB with phosphotungstate/Mg2+; low density lipoprotein cholesterol. Low density lipoprotein cholesterol (LDL-C) levels were estimated using the Friedewald formula as modified by DeLong et al. [23]. HOMA-IR was calculated as the product of the fasting plasma insulin level in microunits and the fasting plasma glucose level in millimoles per liter, divided by 22.5 [24].

Using a radioimmunoanalysis method, glucose and insulin levels were determined while fasting, and 2 hours after the administration of an oral load of 1. 75 g of hydric glucose per kg of body weight, (maximal dose of 75 g). All biochemical indicators, except for post-load glucose and insulin levels, were measured again after 16-week follow-up.

Prior to the start of both diets, the patients and their families were trained how to build a healthy menu based on rations and equivalents. Food replicas were used for instruction and the main food groups included fruits, vegetables, meats, dairy, cereals, legumes, and fats.

Following the first month of instruction, 24 children and adolescents received the Mediterranean-style diet (MSD) and 25 received the standard diet. The Schofield equation was used for dietary calculations in children aged 3–10 and adolescents aged 10–18. The food received by the standard diet group was distributed with 55-60% of carbohydrates (45-50% complex and no more than 10% refined and processed sugars), 25-30% lipids, and 15% proteins. In accordance with the calories corresponding to them, each patient was encouraged to consume fresh fruit and vegetables, while any dairy products and products of animal origin should be low in fat. It was recommended that they limit their consumption of fried food, while the consumption of steamed foods was encouraged. It was suggested that eating times be respected and that food should preferably be consumed at home. It was also suggested that the consumption of fast food be limited to once a month or less. The consumption of sugary drinks and packaged juices was limited, while the consumption of natural water was encouraged along with drinks prepared using fruit but no added sugar. As well as receiving the aforementioned recommendations, the MSD group also received the following specific indications regarding distribution: 60% carbohydrates (50% complex and no more than 10% refined and processed sugars), 25% lipids, and 15% proteins. It was also recommended that they consume foods rich in a) essential fatty acids, such as safflower, corn, olive, and soy oils; b) omega 3 fatty acids (alpha-linolenic acid, eicosapentaenoic acid (EPA), and docosahexaenoic acid (DHA), found in foods such as salmon, mackerel, tuna, grouper, anchovies, flaxseed, canola, walnuts, and wheat germ; c) omega 9 fatty acids (oleic and erucic acids), found in olive and canola oils, as well as Erysimum and mustard seeds; d) antioxidants, such as beta carotenes, lycopenes, vitamin A, vitamin C (found in papaya, strawberries, kiwi, oranges and other citrus fruits, green and red peppers, broccoli, spinach, and raw tomatoes), vitamin E, selenium, zinc, copper, and flavonoids (found in grapes, apples, cherries, broccoli, radishes, beets, seeds, flowers, green tea, black tea, soy, Ginkgo biloba, thistle, and cranberries); and e) fiber (found in fruits and cereals). The MSD group received specific menus including the aforementioned food items. To determine the participants’ typical diet prior to the intervention, a 24-hour recalls was used.

In both groups the diet was administered using a food equivalents system.

For this purpose, food replicas were used so that patients and their parents/guardians could learn to recognize a healthy diet, and menus were tailored according to the age- and gender-required calories of each participant. The patients received their diet plans in writing, containing a graphic representation of the food groups, menu prototypes and general recommendations for healthy nutrition.

The calorie content prior to and after the intervention was calculated using the computer software NUTRIPAC, which was validated in Mexico and contains Hispanic food data [25,26]. Using this software, the calorie content as well as the macro- and micronutrient content of the diets of the intervened patients was determined based on their 24-hour recalls entries.

Both groups received general recommendations about performing physical activity.

Patients were evaluated every three weeks over the intervention to measure diet compliance and reinforce the indicated diet. For this purpose, 24-hour recalls were used. During the consultations, doubts about the indicated diet were resolved, the principles of the intervention were reinforced, barriers/difficulties were discussed, and specific suggestions for better diet compliance were made.

Using the aforementioned technique, measurements were registered during each visit for the following parameters: body composition, dietary assessment, waist and hip circumference measurements, and BP.

Paired Student’s t-test was used for dependent samples to compare the effect of the intervention on the anthropometric, biochemical, and dietary variables in both groups. Macnemar’s test was used to identify the effect of the intervention on cardiovascular risk factors. Data analysis was performed using the SPSS version 20 Package.

## Results

A total of 7 patients (4 from MSD group and 3 from the standard diet) were eliminated from the analysis because they did not attend 90% of the scheduled appointments to assess diet compliance. At the beginning of the intervention, no statistically significant differences were found in sociodemographic, biochemical, clinical, and anthropometric variables, indicating that the study population was homogenously distributed in both groups, as can be observed in Table 1.

**Table 1:** Baseline characteristics of obese children and adolescents according to treatment group

	**Mediterranean style diet n** = **24**	**Standard diet n** = **25**	**p-value**
Male [n (%)]	13 (54)	10 (40)	0.393
Age (years)	11.2 ± 2.7	11.4 ± 2.9	0.737
Obesity onset age (years)	5.58 ± 3.0	4.3 ± 3.2	0.163
Weight (kg)	64.05 ± 10.1	62.2 ± 20.9	0.751
Height (cm)	151.5 ± 12.2	150.5 ± 14.2	0.790
BMI (kg/m^2^)	27.3 ± 3.9	26.7 ± 4.7	0.631
Pre-pubertal [n (%)]	11 (46)	13 (52)	0.778
Schooling (years completed)	5.2 ± 2.3	5.1 ± 2.8	0.949
High BP levels [n (%)]	1 (4)	3(12)	0.609
Acanthosis nigricans [n (%)]	18 (75)	21 (84)	0.496
Low birth weight <2.5 kg [n (%)]	1 (4)	4 (16)	0.603
High birth weight >4.0 kg [n (%)	1 (4)	3 (12)	0.204
HOMA IR ≥ 3 [n (%)]	15 (62)	17 (68)	0.310
Fasting glucose (mg/dL)	98.7 ± 5.5	98.4 ± 5.8	0.849
Glucose 120 minutes (mg/dL)	118.2 ± 16.5	111.3 ± 27.2	0.292
Fasting Insulin (μUI/dL)	18.6 ± 12.8	19.1 ± 12.7	0.896
Insulin 120 minutes (μUI/dL)	107.8 ± 102.9	129.3 ± 142.1	0.548
HOMA-IR	4.5 ± 3.0	4.7 ± 3.3	0.840
Metabolic syndrome [n (%)]	16 (66)	10 (40)	0.060

Data are presented as mean ± SD and frequencies and percentages; the p-value was calculated with Student’s t-test and Chi square test.

**Figure 1:**
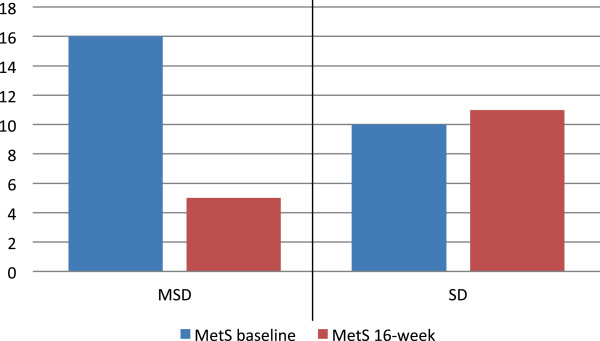
**Changes in metabolic syndrome (MetS) frequency according to diet type.***Mediterranean*-*style diet* (*MSD*) - MetS presented as frequency, difference at baseline and at 16 weeks follow-up was analyzed using McNemar related-samples test (p = 0.003). *Standard diet* (SD)- MetS presented as frequency, difference at baseline and at 16 weeks follow-up was analyzed using McNemar related-samples test (p = 0.999).

The effect of the 16-week intervention on the anthropometric and biochemical variables for both study groups is presented in Table 2. In the MSD group a decrease in the following measurements was observed (p < 0.05): BMI, fat mass, lean mass, glucose, TC. TG, and LDL-C levels. Furthermore, a statistically significant increase in HDL-C was observed in the MSD group, (p < 0.05). The frequency of components of MetS also significantly decreased in the group following the MSD (p < 0.05). With regard to MetS, the group following the MSD showed a decrease of 45% in MetS, and as such, a significant difference after 16-week of intervention, (p < 0.05). The standard diet group decreased the glucose mean levels and the frequency glucose > 100 mg/dL after 16-week intervention (p < 0.05); weight, media arm circumference (MAC) and lean mass increase in the same period of time. This group showed no significant changes in MetS proportion, as can be seen in Figure 1.

**Table 2:** **Differences in cardiovascular risk factors in obese children and adolescents after 16**-**week follow**-**up**

	**Mediterrean style diet**					**Standard diet**				
	**Baseline mean ± SD**	**16-week mean ± SD**	**Δ baseline versus 16-week**		p*	**Baseline mean ± SD**	**16-week mean ± SD**	**Δ baseline versus 16-week**		p*
			**Mean difference**	**95% CI**				**Mean difference**	**95% CI**	
Weight (Kg)	64.0 ± 19.1	63.5 ± 18.6	0.5	-1.8, 0.7	0.369	62.2 ± 20.9	63.9 ± 19.8	-1.7	-2.9, -0.5	0.007
Height (m)	1.51 ± 0.12	1.54 ± 0.11	0.03	0.01, 0.04	0.001	1.50 ± 0.14	1.52 ± 0.12	0.02	0.01, 0.03	0.001
BMI (kg/m^2^)	27.3 ± 3.9	26.2 ± 3.9	-1.10	-1.4, -0.7	0.001	26.7 ± 4.7	26.8 ± 4.5	0.1	-0.2, 0.6	0.374
Waist (cm)	89.0 ± 12.6	88.8 ± 12.7	-0.2	-2.5, 2.0	0.826	84.8 ± 12.2	87.3 ± 11.7	2.5	-0.2, 5.2	0.072
Hip (cm)	97.0 ± 10.9	96.1 ± 10.8	-0.9	-2.8, 0.9	0.303	94.2 ± 12.2	95.2 ± 11.4	1.0	-1.0, 3.0	0.308
Waist/hip ratio	0.913 ± 0.05	0.918 ± 0.06	0.005	-0.015, 0.024	0.600	0.89 ± 0.06	0.91 ± 0.06	0.02	-0.01, 0.04	0.325
MAC (cm)	29.4 ± 4.2	29.7 ± 4.6	0.3	-0.2, 0.9	0.222	28.2 ± 4.7	29.9 ± 3.5	1.7	0.5, 2.7	0.004
TSF (mm)	27.0 ± 7.0	25.5 ± 4.8	-1.5	-3.0, 0.04	0.056	27.9 ± 4.6	28.6 ± 5.0	0.7	-1.2, 2.7	0.444
Fat mass (kg)	26.0 ± 11.2	23.4 ± 10.6	-2.6	-3.4, -1.8	0.001	24.2 ± 9.5	24.5 ± 8.46	0.3	-0.7, 1.3	0.575
Lean mass (kg)	38.0 ± 10.9	40.1 ± 10.9	2.1	0.8, 3.2	0.001	38.0 ± 13.2	39.5 ± 14.0	1.5	0.2, 2.6	0.018
SBP (mmHg)	103.0 ± 11.9	100.3 ± 7.94	-2.7	-7.2, 1.8	0.229	101.2 ± 13.4	100.6 ± 10.7	-0.6	-4.2, 3.0	0.720
DBP (mmHg)	64.0 ± 7.7	62.3 ± 8.6	1.7	-6.0, 2.6	0.435	61.7 ± 9.6	63.2 ± 7.6	1.5	-1.6, 4.6	0.329
Glucose (mg/dL)	98.7 ± 5.5	88.2 ± 3.81	-10.5	-13.1, -7.7	0.001	98.4 ± 5.8	93.5 ± 5.73	-4.9	-8.1, -1.7	0.004
TC (mg/dL)	177.3 ± 24.6	146.3 ± 21.4	-31.0	-42.5, -19.6	0.001	170.2 ± 34.6	166.8 ± 32.3	-3.4	-16.5, 9.7	0.599
TG (mg/dL)	214.4 ± 92.9	124.4 ± 31.1	-90.0	-126.5, -53.4	0.001	197.9 ± 107.3	167.4 ± 78.5	-30.5	-61.9, 1.03	0.057
HDL-C (mg/dL)	34.7 ± 6.3	43.7 ± 9.6	9.0	4.0, 14.0	0.001	31.9 ± 8.5	34.8 ± 12.0	2.9	-2.2, 7.9	0.265
LDL-C (mg/dL)	99.7 ± 26.8	77.7 ± 18.6	-22.0	-33.8, -10.3	0.001	98.7 ± 26.4	98.6 ± 29.7	-0.1	-13.0, 12.7	0.981
	n (%)	n (%)			**	n (%)	n (%)			**
Glucose >100 mg/dL	8 (33.8)	0	-	-	0.007	14 (56)	5 (20)	-	-	0.012
TG >150 mg/dL	21 (88)	2 (8)	-	-	0.001	13 (52)	14 (56)	-	-	0.999
TC > 200 mg/dL	5 (21)	0	-	-	0.025	5 (20)	5 (20)	-	-	0.999
HDL-C ≤ 40 mg/dL	20 (83)	7 (29.2)	-	-	0.002	21 (84)	20 (80)	-	-	0.999
LDL-C >100 mg/dL	10 (42)	4 (17)	-	-	0.058	10 (40)	10 (40)	-	-	0.999
MetS	16 (66.7)	5 (20.8)	-	-	0.003	10 (40.0)	11 (44.0)	-	-	0.999

*MAC*. media arm circumference. *SBP* = systolic blood pressure, *DBP* = diastolic blood pressure, *TC* = total cholesterol, *TG* = triglycerides, *HDL*-*C* = high-density lipoprotein cholesterol, *LDL*-*C* = low-density lipoprotein cholesterol, *CI*. Confidence Intervals *Paired Student’s t-test and **McNemar’s test was used for comparison baseline versus 16-week Δ change magnitude.

**Figure 2:**
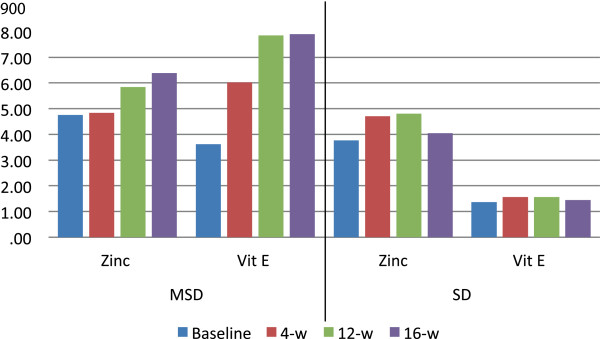
**Dietary consumption of zinc and vitamin E according to the type of diet.***Mediterranean*-*style diet* (*MSD*) – Zinc and vitamin E are expressed as mean (mg), the difference in consumption at baseline and 16 weeks follow-up was analyzed with a paired t test (p = 0.001, p < 0.001 respectively). *Standard diet* (SD) – Zinc and vitamin E are expressed as mean (mg), the difference in consumption at baseline and 16 weeks was analyzed with a paired t test (p = 0.446, p = 0.880 respectively).

**Figure 3:**
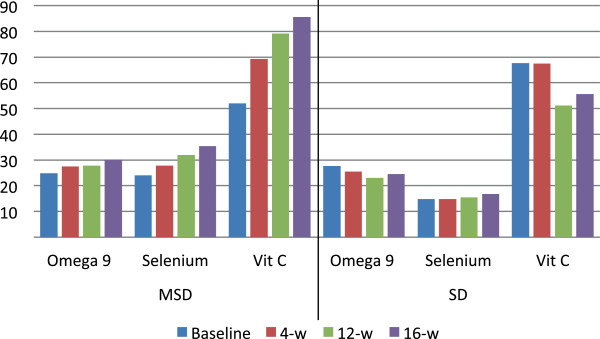
**Dietary consumption of omega 9 fatty-acids, selenium, vitamin C according to the type of diet.***Mediterranean style diet* (*MSD*) – Omega 9 fatty acids (g), selenium (μg), and vitamin C (mg) are expressed as mean, the difference in consumption at baseline and 16 weeks follow-up was analyzed with a paired t test (p = 0.021, p < 0.001, p = 0.037 respectively). *Standard diet* (*SD*) – Omega 9 fatty acids (g), selenium (μg), and vitamin C (mg) are expressed as mean, the difference in consumption at baseline and 16 weeks follow-up was analyzed with a paired t test (p = 0.330, p = 0.490, p = 0.435 respectively).

With respect to diet composition, the MSD group exhibited a significant increase in the consumption of dietary fiber, proteins, omega 9 fatty acids, zinc, selenium, vitamin E, and flavonoids, furthermore, they consumed fewer saturated fatty acids (p < 0.05); No significant changes were identified in the components of the diet in the standard diet group (Table 3).The consumption of zinc, and vitamin E from both diets is shown in Figure 2, it can be observed a greater consumption of these nutriments in the MSD group.Similarly, the changes in dietary consumption of omega 9 fatty acids, selenium, and vitamin C during the 16-week intervention are depicted in Figure 3; the MSD group had a greater consumption of these nutriments.

**Table 3:** **16**-**week diet comparison in obese children and adolescents with any MetS component**

	**Mediterrean style diet**						**Standard diet**				
	**Baseline mean ± SD**	**16-week mean ± SD**	**Δ baseline versus 16-week**		p*	**Baseline mean ± SD**	**16-week mean ± SD**	**Δ baseline versus 16-week**		p*	
			**Mean difference**	**95% CI**				**Mean difference**	**95% CI**		
Energy (Kcal)	1848.9 ± 493.5	1706.7 ± 355.3	-142.1	-322.6, 38.3	0.117	1754.7 ± 570.4	1651.7 ± 367.4	-103.0	-285.2, 79.4	0.255
Fiber (g)	11.0 ± 4.7	16.9 ± 4.85	5.9	3.7, 8.0	0.001	9.10 ± 5.1	8.05 ± 4.1	-1.05	-3.5, 1.5	0.423
Cholesterol (mg)	167.6 ± 60.5	144.9 ± 61.5	-22.6	-49.0, 3.8	0.090	295.9 ± 246.6	210.7 ± 206.9	-85.2	-237.2, 66.8	0.259
CHO (g)	230.3 ± 72.4	203.1 ± 33.9	-27.2	-57.3, 2.9	0.075	204.5 ± 93.4	197.7 ± 72.3	6.8	-30.6, 17.0	0.562
CHO (%)	49.5 ± 6.8	48.0 ± 4.4	-1.5	-4.6, 1.6	0.428	45.8 ± 10.2	47.0 ± 9.9	1.2	-2.8, 5.2	0.551
Proteins (g)	69.3 ± 18.4	72.3 ± 11.5	3.0	-4.9, 10.8	0.449	60.9 ± 16.8	58.2 ± 17.0	-2.7	-9.8, 4.5	0.458
Proteins (%)	15.2 ± 3.0	17.1 ± 2.1	1.9	0.4, 3.4	0.012	14.3 ± 3.1	14.0 ± 3.3	-0.3	-1.4, 0.9	0.688
Lipids (%)	35.6 ± 6.4	35.1 ± 4.8	-0.5	-3.3, 2.3	0.719	39.9 ± 9.4	38.9 ± 9.4	-1.0	-5.1, 3.2	0.640
SFA (%)	30.2 ± 3.9	27.7 ± 4.2	-2.5	-4.6, -0.3	0.024	34.4 ± 9.6	33.1 ± 7.5	-1.3	-5.7, 3.1	0.556
MUFA (%)	41.1 ± 2.7	42.3 ± 3.8	1.2	-0.2, 2.7	0.094	40.4 ± 5.7	40.0 ± 4.2	-0.4	-2.5, 1.8	0.742
PUFA (%)	28.7 ± 4.0	29.9 ± 4.2	1.2	-0.4, 2.9	0.144	25.2 ± 5.6	26.8 ± 5.9	1.6	-1.4, 4.7	0.292
Omega 9 FA (g)	24.9 ± 10.4	30.2 ± 12.4	5.3	0.8, 9.7	0.021	27.7 ± 12.9	24.4 ± 9.9	-3.3	-9.9, 3.4	0.330
Sodium (mg)	1418.8 ± 367.2	1326.4 ± 432.6	-92.33	-270.0, 85.3	0.294	1297.4 ± 578.7	1182.9 ± 553.5	-114.5	-366.0, 137.0	0.357
Potassium (mg)	1614.8 ± 515.5	1545.1 ± 374.0	-69.6	-257.3, 118.2	0.007	1491.6 ± 599.7	1565.9 ± 656.6	74.3	-168.7, 317.3	0.534
Zinc (mg)	4.7 ± 1.4	6.4 ± 2.5	1.7	0.7, 2.5	0.001	3.7 ± 1.65	4.0 ± 2.03	0.3	-0.4, 1.0	0.446
Selenium (μg)	24.0 ± 8.5	35.4 ± 8.4	11.4	6.6, 16.0	<0.001	14.9 ± 18.2	16.7 ± 11.65	1.8	-3.5, 7.2	0.490
Vitamin C (mg)	52.0 ± 40.5	85. 6 ± 79.7	33.6	2.1, 64.8	0.037	67.6 ± 62.9	55.5 ± 70.9	-12.1	-43.4, 19.2	0.435
Vitamin E (mg)	3.6 ± 2.8	7. 9 ± 2.9	4.3	2.6, 5.8	<0.001	1.44 ± 2.2	1.37 ± 2.6	0.07	-0.93, 1.08	0.880
Flavonoids (mg)	12.4 ± 5.1	20.2 ± 5.4	7.8	5.4, 10.0	<0.001	2.9 ± 4.25	2.7 ± 4.1	-0.2	-2.2, 1.7	0.819

*Kcal* = kilocalories, *g* = grams, mg = milligrams, *CHO* = carbohydrates, *FA* = fatty acids, *SFA* = saturated fatty acids, *MUFA* = monounsaturated fatty acids, *PUFA* = polyunsaturated fatty acids. *CI* = Confidence Intervals *Paired Student’s t-test was used for comparison baseline versus 16-week. Δ change magnitude.

## Discussion

The results of the present study demonstrate the effect of a nutritional intervention with an MSD in obese children exhibiting at least one MetS component. The benefits of a Mediterranean diet on health, cardiovascular disease prevention, and other aliments have been shown in adult populations [27,28]. The characteristics of our study participants, who were obese and exhibited at least one cardiovascular risk factor, made them ideal candidates for the implementation of a diet aimed at decreasing these factors. The intervention consisted of the administration of a nutrition plan containing foods that are part of a Mediterranean diet.

Although the benefit of a Mediterranean diet in adults is widely recognized, little is known about its effect in children. Studies have reported an association between diet compliance and obesity prevalence in children and adolescent populations [29].

Even though adherence to the Mediterranean diet is important, other factors also influence obesity prevention, such as eating habits, customs, and physical exercise [30]. Thus, it is necessary to strengthen adherence to the Mediterranean diet while also addressing other factors that contribute to obesity and metabolic alterations.

After a 16-week intervention, the MSD group decreased BMI but without changes in weight. Lopez Alarcon et al., who conducted an intervention study supplementing 900 mg omega 3 fatty acids to a population aged 9 to 18 years, they reported not changes in weight or BMI [31]. Similarly Fernandez et al., who compared three different diets over a three-month period in an adult population and did not observe an association between body weight and the Mediterranean diet [32]. Although the MSD had no significant effect on body weight after four months of intervention, a decrease in BMI and an increase in height were observed. The nutrition plan was administered according to weight, sex, age, and eating habits and was not intended to be a weight reductive treatment. This could explain why the population did not experience any weight loss. On the contrary, the standard diet group increased 1.7 kg average, with no effect on the BMI, possibly due to the height increase.

It could be explained by the dietetic patterns, the lack of physical activity, and the sedentary behavior of studied subjects. Our results agree with those that have reported nutritional and physical activity and motivational interviewing training interventions in similar populations [33,34].

Abdominal obesity was measured by WC, which has been reported to be a strong indicator of cardiometabolic risk in children and adolescents [35,36]. We did not observe a significant change in this indicator at the end of the intervention. It has been reported that higher scores for diet compliance are inversely associated with WC and waist-height ratio in young populations [37]. We measured body composition using bioelectrical impedance and did observe significant differences in the reduction of fat mass and lean mass in the group with MSD. Changes in these indicators have been reported in the Mediterranean diet adherence [38].

High BP levels are an important component of MetS. A total of 4 patients with high BP levels were identified at the beginning of the study. No significant changes in the average SBP and DBP values were found, unlike what has been reported previously for young populations following the Mediterranean diet, albeit with higher compliance rates [39]. One possible explanation could be that our study population already exhibited age- and sex-appropriate BP values.

Although the development of T2DM is still more common in adults, an increase in this disease has been reported in children and adolescents [40]. Although genetic factors predispose individuals to the development of diabetes, obesity, lifestyle, poor living conditions, and social factors further contribute to it [41]. In Mexico, a 0.6% prevalence of diabetes in the pediatric population has been reported, which further increases to 1.3% in obese children [42]. In our study population, 8 participants receiving the MSD and 14 participants receiving the standard diet presented with glucose values greater than 100 mg/dL. This is consistent with previous reports that obesity continues to be a determining factor in fasting glucose alterations and IR [43].

In the present study, we observed a decrease in fasting glucose levels in both intervention groups. However, the MSD group exhibited the most significant decrease. A reduction in simple sugar consumption was suggested for both diets; thus, a possible explanation for this observation could be that the MSD group had a higher fiber content, which could have a greater influence on reducing glucose levels. Furthermore, 65% of the total assessed population presented with IR, assessed by HOMA-IR >3, which is similar to what has been previously reported by other groups [44]. Importantly, an inadequate lifestyle leads to a higher risk of developing T2DM.

The results of the current study demonstrated that MSD decrease CT, TG and LDL-C levels and also increase HDL-C after the 16-week. Similar results have been previously reported in obese children and adolescents following a hypocaloric diet low in simple carbohydrates and high in vegetables and fiber-rich cereals [43]. Other authors have reported in children with hypercholesterolemia decrease in TC and LDL-C levels with a Mediterranean diet [45].

The results are relevant in regard to these indicators, given that alterations in the lipid profile are recognized as an important factor in cardiovascular risk among the overweight and obese pediatric population [44]. In the standard diet group no significant changes were observed in lipid profile.

One of the characteristics of the administered MSD was its high fiber content, which is most likely responsible for the reduction in glucose and lipid levels, as mentioned previously [46]. Similar effects on metabolic indicators have been reported for pre-diabetic Mexican adults following a diet with a 50% reduction in carbohydrates and higher fruit, vegetable, and fiber content [47] The fiber consumption in the MSD group was greater than 16 g. This is similar to the value reported for a population on a diet with many food items typical of a Mediterranean diet [48]. The MSD group increased significantly the fiber consumption, this is beneficial, since it has been reported the association between a low fiber diet and higher adiposity and cardiovascular risk factors during childhood and adolescence [49,50]. Another benefit of the MSD was the increased protein intake, this is relevant because these nutrients are essential for a healthy growth and development in this population.

One characteristic of the MSD in this study was the greater consumption of antioxidants, such as selenium, vitamins C and E, dietary fiber and few simple sugars, this could explain that MSD group exhibited a higher significantly average consumption of omega 9 fatty acids, dietary fiber and less consumption of SFA, different to what we found in the standard diet group.

MSD diet group exhibited important benefits in reducing several MetS components, which is consistent with the decrease in metabolic risk indicators reported in women following a diet supplemented with antioxidants [51]. MetS was identified in a significant proportion of both groups. However, at the end of the intervention, the MSD group showed a significantly reduced proportion of patients exhibiting MetS in regard to the basal metabolic rate. As previously reported, our results support the usefulness of providing children exhibiting obesity and MetS with an MSD aimed at increasing consumption of fiber, proteins, omega 3 and omega 9 fatty acids, zinc, selenium, vitamin E and flavonoids [15].

These results further support the importance of introducing an MSD to at-risk populations. In addition, a healthy lifestyle, adequate eating habits, and physical activity should be encouraged, while less time should be spent watching TV, working at the computer, and playing video games. A lack of physical activity has been reported to be a major predicting factor of childhood obesity, superseding compliance with a Mediterranean diet [52]. Physical activity was not evaluated in this study, both groups received general recommendations in this area. Future research should be carried out to evaluate the efficacy of combining a MSD with physical activity in childhood obesity.

A limitation of our study was the short intervention time, thus, the sustainability of longer intervention time should be assessed. We also consider that this strategy should be evaluated with a larger number of participants to adjust the effect by variables such as age, socioeconomic status, physical activity and sedentary status. Our results suggest that in the Mexican population, a longer intervention time with an MSD, together with lifestyle modifications could have a greater impact on the pediatric population exhibiting obesity and at least one MetS component.

## Conclusions

An MSD improves the BMI, glucose, and lipid profile in children and adolescents exhibiting obesity and any MetS component.

## Abbreviations

MetS: Metabolic syndrome; IR: Insulin resistance; TG: Triglycerides; HDL-C: High density lipoprotein cholesterol; T2DM: Type 2 diabetes mellitus; GI: Glycemic index; GL: Glycemic load; HOMA-IR: Homeostatic Model Assessment of Insulin Resistance; BP: Blood pressure; TC: Total cholesterol; MSD: Mediterranean-style diet; BMI: Body Mass Index; WC: Waist circumference; IDF: International Diabetes Federation; SBP: Systolic blood pressure; DBP: Diastolic blood pressure; LDL-C: Low density lipoprotein cholesterol; EPA: Eicosapentaenoic acid; DHA: Docosahexaenoic acid; TSF: Tricipital skinfold.

## Competing interests

As authors of this manuscript, We state that there is no competing interests of any particular form, and that this research was carried out with the financial support provided by the Coordination of Health Research belonging to the Mexican Institute of Social Security.

## Authors’ contributions

L. Velazquez, GSy JN designed the study and carried out the experiments. LV, MT, AM and PM performed the statistical analysis and contributed to the critical revision of the manuscript. All authors contributed to drafting the manuscript and all authors read and approved the final manuscript.

## Authors’ information

L Velazquez is MSc., and Ph.D. Student (She works in a Health Research Unit) involved with Metabolic Disorders Research. J Nava and G Santiago are dieticians involved in metabolic disorders. A Muñoz is MSc., Ph.D. Candidate with emphasis in Epidemiology, Adjunct Professor of Public Health, involved in clinical trials. P Medina is MD, Ph.D., involved in Pediatric Metabolic Disorders Research. M Torres is MD, Ph.D., involved in health programs related to metabolic disorders in Mexican childhood.

## Pre-publication history

The pre-publication history for this paper can be accessed here:

http://www.biomedcentral.com/1471-2431/14/175/prepub
